# Editorial: Biosynthesis of Amino Acids and Their Derived Chemicals From Renewable Feedstock

**DOI:** 10.3389/fbioe.2021.770002

**Published:** 2021-10-13

**Authors:** Yi-Rui Wu, K. Madhavan Nampoothiri, Congqiang Zhang, Liming Liu

**Affiliations:** ^1^ Department of Biology, Shantou University, Shantou, China; ^2^ Beijing Tidetron Bioworks Company, Beijing, China; ^3^ National Institute for Interdisciplinary Science and Technology (CSIR), Thiruvananthapuram, India; ^4^ Singapore Institute of Food and Biotechnology Innovation (SIFBI), Agency for Science, Technology and Research, Singapore, Singapore; ^5^ State Key Laboratory of Food Science and Technology, Jiangnan University, Wuxi, China

**Keywords:** amino acids, biomanufacturing, metabolic engineering, microbial fermentation, renewable feedstock

This research topic aimed to introduce the current advancements in the biosynthesis of amino acids and their derived products from renewable feedstocks. Authors were invited to contribute original research and review articles that provided a comprehensive discussion and analysis of the current success and future outlooks for biosynthesis of various amino acids and derived chemicals. A total of 12 manuscripts were submitted, and 11 were accepted for publication after a thorough and rigorous peer review process. The papers were selected in such a way to give a flavor of a variety of topics related to the production of amino acids and their derived products, including biological and chemical catalytic production, design and construction of new molecular pathways for amino acid production, application of metabolic engineering and synthetic biology strategies, as well as the utilization of non-food renewable feedstock such as rice straw. We believed that the papers in this research topic would bring readers the latest advances in these fields.

Bioproduction of 5-aminovalerate (5AVA) from renewable feedstock could support a sustainable biorefinery process to produce bioplastics. The paper by Cheng et al. developed a promising artificial pathway for the efficient 5AVA synthesis by establishing a 2-keto-6-aminocaproate-mediated pathway. Introduction of l-lysine α-oxidase from *Scomber japonicas*, α-ketoacid decarboxylase from *Lactococcus lactis* and aldehyde dehydrogenase from *Escherichia coli* could finally achieved the biosynthesis of 5AVA from l-lysine with the high titre through the fed-batch fermentation. Another paper by Cheng et al. further presented an efficient biobased co-production of 5AVA and δ-valerolactam in *E. coli* from l-lysine. With the optimized cultivation conditions, the titers of 5AVA and δ-valerolactam were improved, and their ratio was identified to be affected by pH values. The paper by Sasikumar et al. established the production of 5AVA and putrescine from the biomass-derived sugars by using the engineered *Corynebacterium glutamicum* strain. It was indicated that with the heterologous introduction of genes xylA_Xc_ and xylB_Cg_, the modified strain could co-produce putrescine and 5-AVA by consuming a blend of glucose and xylose. Further investigation by using alkali-hydrolases pretreated rice straw hydrolysate (RSH) as the raw material also yielded the generation of putrescine and 5AVA. The paper by Brito et al. demonstrated another study involved in the methanol-based production of 5AVA using genetically modified *Bacillus methanolicus*. Five different metabolic pathways were evaluated, whereof two directly converted l-lysine to 5AVA and three used cadaverine as an intermediate. The results indicated the proof-of-concept 5AVA production from methanol at 50°C, enabled by two pathways out of the five tested with the highest titer, representing the first report of 5AVA production from methanol in the methylotrophic bacteria.

Protocatechuic acid (PCA) was a strong antioxidant and could also be used as a potential platform for polymer building blocks. The paper by Englund Örn et al. presented the production of PCA from glucose through the shikimate pathway by heterologously expressing 3-dehydroshikimate dehydratase encoded gene in *E. coli*. With the overproduction of phenylalanine to relieve the allosteric inhibition of 3-deoxy-7-phosphoheptulonate synthase by the aromatic amino acids, the engineered strain was shown to achieve a highest PCA yield during cultivation in fed-batch mode using a feed of glucose and ammonium salt. As another trifunctional building block, L-2-hydroxyglutarate (L-2HG) was also highly attractive for the chemical and pharmaceutical industries. The paper by Prell et al. demonstrated the natural biosynthesis of L-2HG by metabolically engineering *C. glutamicum* through the construction of a gene cassette involved in a six-step synthetic pathway. The modified strain with media adaptation was observed to produce L-2HG in a micro-cultivation system, and a high titer of L-2HG was finally achieved via a glucose-based process in a 2 L bioreactor.

l-Carnitine was a bioactive compound derived from l-lysine and S-adenosyl-l-methionine. The paper by Kugler et al. described the metabolic engineering of *E. coli* for l-carnitine production by introducing and optimizing a four-step pathway from the fungus *Neurospora crassa*. The engineered strain was investigated to produce l-carnitine by supplementing L-Nε-trimethyllysine for biotransformation. The work provided a proof of concept as the first report for the *de novo* l-carnitine production in the engineered bacteria.

The paper by Ren et al. presented *Saccharopolyspora erythraea* as an excellent host to produce valuable heterologous polyketides. The recombinant strain AbΔery was genetically generated by knocking out the erythromycin biosynthesis gene cluster via the CRISPR-Cas9 system, and three heterologous genes driven by strong promoters were subsequently introduced to produce novel polyketide by using l-tyrosine and methylmalonyl-CoA as the substrates. With the final product of (E)-4-hydroxy-6-(4-hydroxystyryl)-3,5-dimethyl-2H-pyrone identified by LC-MS, the engineered strain Ab∆ery could potentially serve as a precious heterologous host to boost the synthesis of other valuable polyketone compounds.

The paper by E et al. investigated the effect of biochar on an enhanced production of l-histidine from glucose by *E. coli*. The optimal biochar concentration was demonstrated, and too high-concentration treatment was found to inhibit the yield. A regulatory protein (HisG) was further identified to be responsible for the improved l-histidine production through the protein docking analysis and gene overexpression.

*Corynebacterium glutamicum* was a model Gram-positive bacterium that had been extensively engineered to produce amino acids and other chemicals. The review paper from Zhang et al. summarized the recent progress on the metabolic engineering of *C. glutamicum* towards various chemicals production, and also discussed potential manners to broaden its substrate spectrum to those non-food sustainable carbon sources such as xylose, methanol, arabinose, glycerol, etc. The review paper from Liu et al. also provided examples to illustrate the latest progress on the microbial synthesis of 5-hydroxytryptophan (5-HTP) in the manner of the directed evolution and metabolic engineering.

